# Rare case of primary spinal ependymomatosis occurring in a 26-year-old man: a case report

**DOI:** 10.1186/1752-1947-3-72

**Published:** 2009-10-12

**Authors:** Chandrasekaran Kaliaperumal, Nigel Suttner, Brian Herron, Kishor A Choudhari

**Affiliations:** 1 National centre for Neurosurgery, Beaumont Hospital, Dublin-9, Republic of Ireland; 2 Department of Neurosurgery, Regional Neurosciences Unit, Royal Victoria Hospital, Belfast BT12 6BA, UK; 3 Department of Neuropathology Regional Neurosciences Unit, Royal Victoria Hospital, Belfast BT12 6BA, UK

## Abstract

**Introduction:**

The authors report a rare case of primary spinal ependymomatosis in a young adult man. Multiple primary ependymomatous lesions were seen on magnetic resonance imaging and no anaplasia was identified on the surgical-pathological analysis. The aetio-pathological mechanism and surgical significance of this rare occurrence is discussed.

**Case presentation:**

A 26-year-old man of Polish origin presented with a ten-day history of pain in the left leg and lower back. This was followed by difficulty in urinating and a decrease in sensation in both legs. Examination revealed pyramidal signs and mild weakness in both lower limbs. He had early sphincter involvement requiring catheterization. Magnetic resonance imaging of the brain was normal. However, that of the spinal cord revealed multiple intradural spinal lesions, both intra- and extramedullary, extending from the cervical cord down to the cauda equina roots. T12-L1 laminectomy was performed. Multiple intradural, extra- and intra-medullary tumors were seen. After the operation, the patient deteriorated with a sensory level at T4. Post-operative cranio-spinal radiotherapy was administered but there was no clinical improvement in the lower limbs.

**Conclusion:**

Primary spinal ependymomatosis is a rare phenomenon involving multiple spinal segments in the absence of a primary intracranial tumor. Radical excision is unrealistic in this condition. Biopsy followed by radiotherapy is the preferred method of treatment.

## Introduction

Ependymomas are usually solitary lesions that may metastasize within the central nervous system (CNS) via the cerebrospinal fluid (CSF) pathways. We describe a rare case of primary spinal ependymomatosis and discuss its clinical presentation and etio-pathogenesis, highlighting issues related to its management.

## Case presentation

A 26-year-old man of Polish origin, previously fit and healthy, presented with a ten-day history of pain in the left leg and lower back. This was followed by difficulty in urinating and decrease in sensation in both legs. Examination revealed pyramidal signs and mild weakness of both lower limbs. He had early sphincter involvement requiring catheterization.

**Figure 1:**
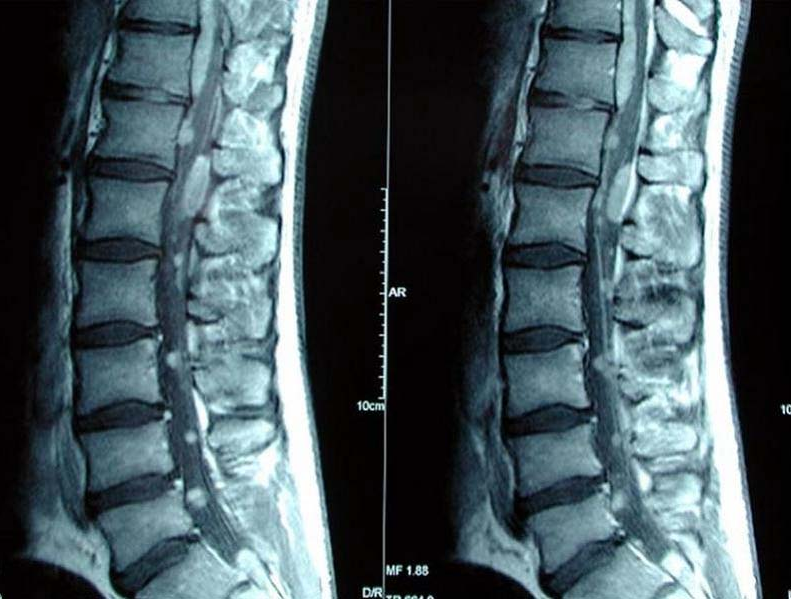
**Sagittal magnetic resonance imaging (MRI) scans of the lumbo-sacral spine showing multiple ependymomatous lesions**.

Magnetic resonance imaging (MRI) of the brain was normal. However, that of the spinal cord revealed multiple intradural spinal lesions, both intra- and extramedullary, extending from the cervical cord down to the cauda equina roots (Figure 1). There was also a syrinx extending from T1 to T10. There were no intracranial lesions.

**Figure 2:**
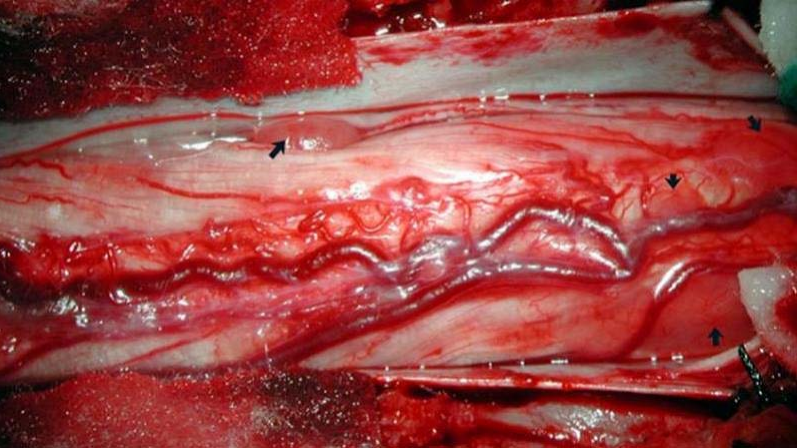
**Intraoperative photograph of multiple ependymomas in the region of conus medullaris and cauda equina (arrow)**.

**Figure 3:**
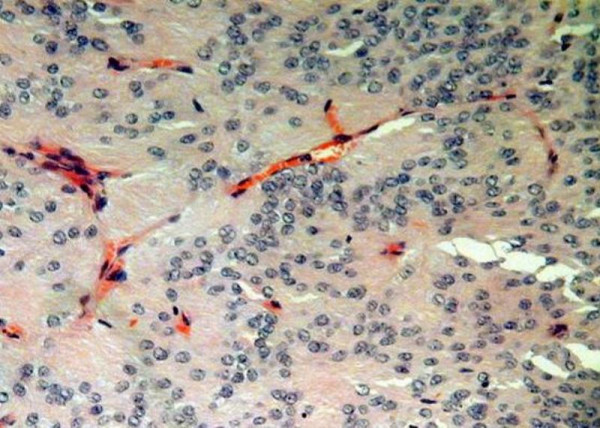
**Histological appearances of a representative ependymomatous tumor**.

A T12-L1 laminectomy was performed. Multiple intradural, extra- and intramedullary tumors were seen (Figure 2). An exophytic intramedullary tumor arising from the conus was biopsied which was confirmed to be an ependymoma. Histology showed a moderately cellular tumor with typical perivascular rosettes. Ki-67 showed a low and/or moderate cell turnover, suggestive of primary typical ependymoma. There was no evidence of anaplastic change (Figure 3). After the operation, the patient deteriorated with a sensory level at T4. Post-operative cranio-spinal radiotherapy was administered, but there was no clinical improvement in the lower limbs.

## Discussion

Spinal ependymomas are rare tumors constituting 1.9% of all CNS tumors and 60% of spinal gliomas [1]. They are commonly seen during the fourth decade of life, and occur more frequently among males, with the majority of cases located in the lumbo-sacral region [1]. Usually intradural, they can be intra- or extramedullary. The conus medullaris and the cauda equina are the most commonly involved sites (95%) of primary spinal ependymomas [1]. Pathologically there are four types of ependymomas, namely: typical, anaplastic, subependymoma and myxopapillary. Among these, mainly anaplastic ependymomas are known to spread tp multiple sites in the spine and cranial cavity [2,3]. Rare cases of tumor spread from the spine retrograde to the cranium [4] and extraneural spread to liver and lungs from the spine have been reported in literature [5].

Primary ependymomatosis is a rare phenomenon where the ependymomas are seen particularly involving multiple segments of the spine without any evidence of a primary intracranial lesion. In most cases of secondary ependymomatosis, multiple involvement of the spine is a result of drop metastases from a cranial or more proximal primary lesion. In our case, we could not determine if the ependymomatosis represented primary synchronous multiple ependymomas, or whether they were simply drop metastases from an asymptomatic cervical primary lesion. In this case, no definitive primary cranial lesion was identified. Also, there was very little anaplasia detected on histopathology. Therefore, we believe that this case represents true primary ependymomatosis of the spinal cord, and not simply a case of drop metastases. Histopathology was suggestive of a primary typical ependymoma and the multiplicity of the lesions in the spinal cord without cranial involvement implies the possibility of this rare phenomenon. In any event, multiplicity of the lesions meant that radical resection with a curative intention was an unrealistic goal. As predominant symptoms and compression were located at the conus level, limited decompression with biopsy of one of the compressive exophytic lesions was performed with no clinical benefit.

Although primary surgical excision is the treatment of choice in most cases of spinal ependymomas, from our experience and in cases of primary ependymomatosis, radical surgical extirpation is not advised. A biopsy of the most accessible representative lesion followed by radiotherapy perhaps represents to be the most preferable option.

## Conclusion

Primary spinal ependymamatosis is a rare phenomenon involving multiple spinal segments without primary intracranial tumor. Radical excision, although recommended for ependymomas in general, is unrealistic and carries high risks of further neurological deterioration. Biopsy followed by radiotherapy may be the most preferred management of this rare condition.

## Abbreviations

CNS: central nervous system; CSF: cerebrospinal fluid; MRI: magnetic resonance imaging.

## Competing interests

The authors declare that they have no competing interests.

## Authors' contributions

CK collected data and prepared the draft for the manuscript. NS prepared the discussion for the case report. BH is a neuropathologist and was involved in tissue analysis and slide preparation for this patient. KAC is the senior neurosurgeon involved in this overall management of the patient. KAC revised the manuscript. All authors read and approved the final manuscript.

## Consent

Written informed consent was obtained from the patient for the publication of this case report and any accompanying images. A copy of the written consent is available for review by the Editor-in-Chief of this journal.
